# Pkm2 can enhance pluripotency in ESCs and promote somatic cell reprogramming to iPSCs

**DOI:** 10.18632/oncotarget.20685

**Published:** 2017-09-06

**Authors:** Shengtang Qin, Danli Yang, Kang Chen, Haolan Li, Liqiang Zhang, Yuan Li, Rongrong Le, Xiaojie Li, Shaorong Gao, Lan Kang

**Affiliations:** ^1^ Institute of Cancer Stem Cell, Dalian Medical University, Dalian 116044, China; ^2^ School of Life Sciences and Technology, Tongji University, Shanghai 200092, China; ^3^ College of Stomatology, Dalian Medical University, Dalian 116044, China

**Keywords:** Pkm2, ESC, iPSC, reprogramming, pluripotency

## Abstract

Aerobic glycolysis is one of the most important common characteristics in both cancer cells and stem cells. Metabolism switch has been discovered as an important early event in the process of reprogramming somatic cells to induced pluripotent stem cells (iPSCs). As a rate limiting kinase in glycolysis, Pkm2 has been reported playing critical roles in many tumors, yet its role in stem cells and iPSCs induction is poorly defined. In the present study, we showed that Pkm2 is a predominant pyruvate kinase in embryonic stem cells (ESCs), and its expression increases many pluripotent genes. During somatic cell reprogramming, up-regulation of Pkm2 can be observed and over-expression of Pkm2 can facilitate iPSCs induction, while Pkm1 or a mutant form of Pkm2 (Pkm2^K422R^) showed no enhancement role in iPSCs induction. Therefore, our data demonstrated that Pkm2 enhances the pluripotency maintenance in ESCs and promotes the pluripotency acquisition during somatic cell reprogramming.

## INTRODUCTION

By ectopically expressing Oct4, Sox2, Klf4 and c-Myc, somatic cells can be reprogrammed into induced pluripotent stem cells (iPSCs) [1]. Exploring the molecular mechanisms involved in reprogramming is attracting great interests, as it will address fundamental questions such as cell identity and cell fate decision and in turn advance iPSCs application. It has been suggested that reprogramming is a multi-phase process, and metabolism switch serves as one of the important early events in reprogramming [2–4]. Indeed, embryonic stem cells (ESCs) possess distinct metabolic features compared with somatic cells [5]. Somatic oxidative bioenergetics transition into aerobic glycolysis is participated and facilitates reprogramming [6].

Aerobic glycolysis has been extensively studied in many tumors. Cancer cells maintained highly glycolysis activity even in the aerobic environment called Warburg effect, thus to generate ATP faster and provide more building blocks to meet the anabolic demands of higher proliferation [7]. Accordingly, the enzymes in the glycolysis were found enriched and participated in numerous tumors’ progression [8, 9]. Catalyzing the transfer of a phosphate group from phosphoenolpyruvate (PEP) to ADP producing pyruvate and ATP, which is a rate-limiting step in glycolysis, Pkm2 has been found highly express in aggressive tumors and play important roles in tumor metabolism, growth and migration [10–14]. Distinct from its splicing analogue Pkm1, Pkm2 was found prefer the lower activity dimer form, which could facilitate the accumulation of semi-products for anabolic demands in tumor cells [15]. And the switch from Pkm1 to Pkm2 was reported crucial in some tumors although controversial in others [16–18]. It is interesting to elucidate the role of Pkm2 in normal tissues especially ESCs which share many similar features to tumor cells.

Pkm2 was known to abundant in proliferating cells, embryonic tissues, and stem cells besides tumor cells [19]. But its function is far from clear in embryonic tissues and stem cells. Here we investigated the performance and function of Pkm1 and Pkm2 in ESCs and somatic cell reprogramming. Our results suggested that Pkm2 is the predominant form in ESCs and plays promoting roles in reprogramming, which would be correlated with its conformation.

## RESULTS

### Pkm2 is the predominant pyruvate kinase in ESCs

**Figure 1:**
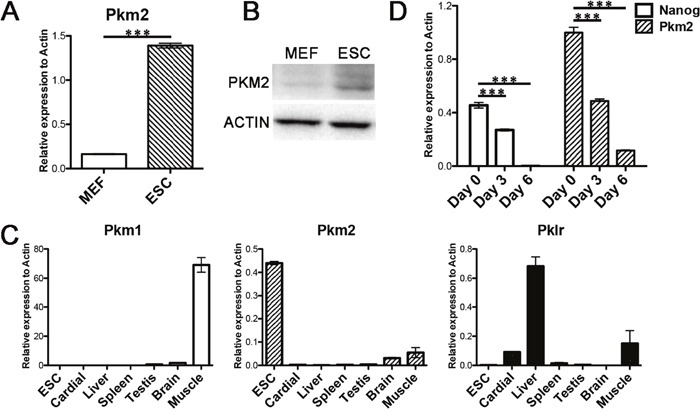
Pkm2 was predominantly expressed in ESCs **(A)** RNA level of Pkm2 in MEFs and ESCs. **(B)** Protein level of Pkm2 in MEFs and ESCs. **(C)** Real-time PCR examining the expression of Pkm1, Pkm2 and Pklr in the indicated tissues. **(D)** Real-time PCR examining the expression changes of Nanog and Pkm2 at the indicated time point during the differentiation of ESCs. All statistical analyses are unpaired Student's t tests, and significance is annotated as not significant (ns), ^*^p ≤ 0.05, ^**^p ≤ 0.01, or ^***^p ≤ 0.001. Data represented as mean ± SD; n = 3.

It has been shown previously that Pkm2 was highly expressed in embryonic tissue and cancer cells [19]. Here, we first examined the expression status of Pkm2 in ESCs and found that Pkm2 showed much higher expression level in ESCs than in mouse embryonic fibroblasts (MEFs) at both mRNA and protein levels (Figure 1A and 1B). Then we examined Pkm1 and Pklr, which are family members of Pkm2, and found that only Pkm2 was enriched in ESCs while Pkm1 expressed predominantly in brain and Pklr expressed in liver (Figure 1C). Then we performed differentiation of ESCs through embryoid body (EB) formation to examine the expression dynamics of Pkm2. As we showed, the expression of Pkm2 decreased along with ESCs differentiation which is similar to the pluripotent gene Nanog (Figure 1C). Therefore, in the pyruvate kinase protein family, Pkm2 was the predominant kinase expressed in ESCs and showed dramatic decreased expression along with the differentiation.

### Overexpression of Pkm1 and Pkm2 can enhance pluripotent genes expression in ESCs

**Figure 2:**
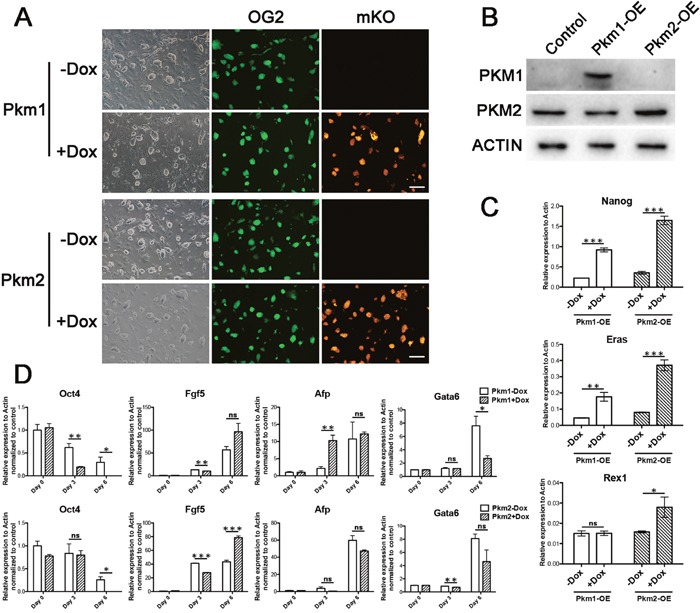
Overexpression of Pkm1 and Pkm2 enhanced pluripotent genes in ESCs **(A)** Morphology of the ES cell lines with Dox inducible overexpression of Pkm1 (upper) or Pkm2 (lower). EGFP indicating the activity of distal enhancer of endogenous Oct4. mKO exhibiting the ectopic expression of Pkm1 or Pkm2 under Dox induction. **(B)** Western blot showing the overexpression of Pkm1 and Pkm2 in established ES cell lines. **(C)** Real-time PCR examining the expression changes of Nanog, Eras and Rex1 under Pkm1 or Pkm2 overexpression in ESCs. **(D)** Expression changes of Oct4, Fgf5, Afp and Gata6 on Day 0, Day 3 and Day 6 during the differentiation of Pkm1 (upper) or Pkm2 (lower) overexpression ES cell lines. Scale bars represent 200 μm. Data represented as mean ± SD; n = 3.

To explore the function of Pkm1 and Pkm2 in ESCs, we first performed the overexpression studies in ESCs by establishing the stable cell lines. We employed the rtTA-OG2 ESCs derived from embryos mating from Gt(ROSA)26Sor^tm1(rtTA^*^M2)Jae^ (rtTA) and Tg(Pou5f1-EGFP)2Mnn (OG2) mice. Doxycycline (Dox) controlled inducible lentiviral overexpression vectors carrying Pkm1, Pkm2 or empty vector tagged with fluorescence protein monomeric Kusabira orange (mKO) was introduced into the rtTA-OG2 ESCs in which EGFP signal indicating the expression of endogenous Oct4. We established stable cell lines with normal colony appearance and Oct4-EGFP positive signal (Figure 2A), which exhibited remarkably increase of the overexpressed genes under the Dox addition indicated by mKO signal and western blot (Figure 2A and 2B). Under the Pkm1 or Pkm2 overexpression, several important pluripotent genes increased including Nanog, Eras and Rex1, and the influence by Pkm2 overexpression was much higher (Figure 2C). Then we wondered if the ectopic expression of Pkm1 or Pkm2 affects the differentiation of ESCs, so we performed EB formation assay. We found that under the overexpression of Pkm1 and Pkm2, ESCs could form EBs normally along with the similar decreasing of pluripotent gene and increasing of differentiation associated genes (Figure 2D). Thus, overexpression of Pkm1 or Pkm2 could enhance pluripotent genes’ expression in ESCs without affecting the differentiation of ESCs.

### Knockdown of Pkm affected pluripotent genes

**Figure 3:**
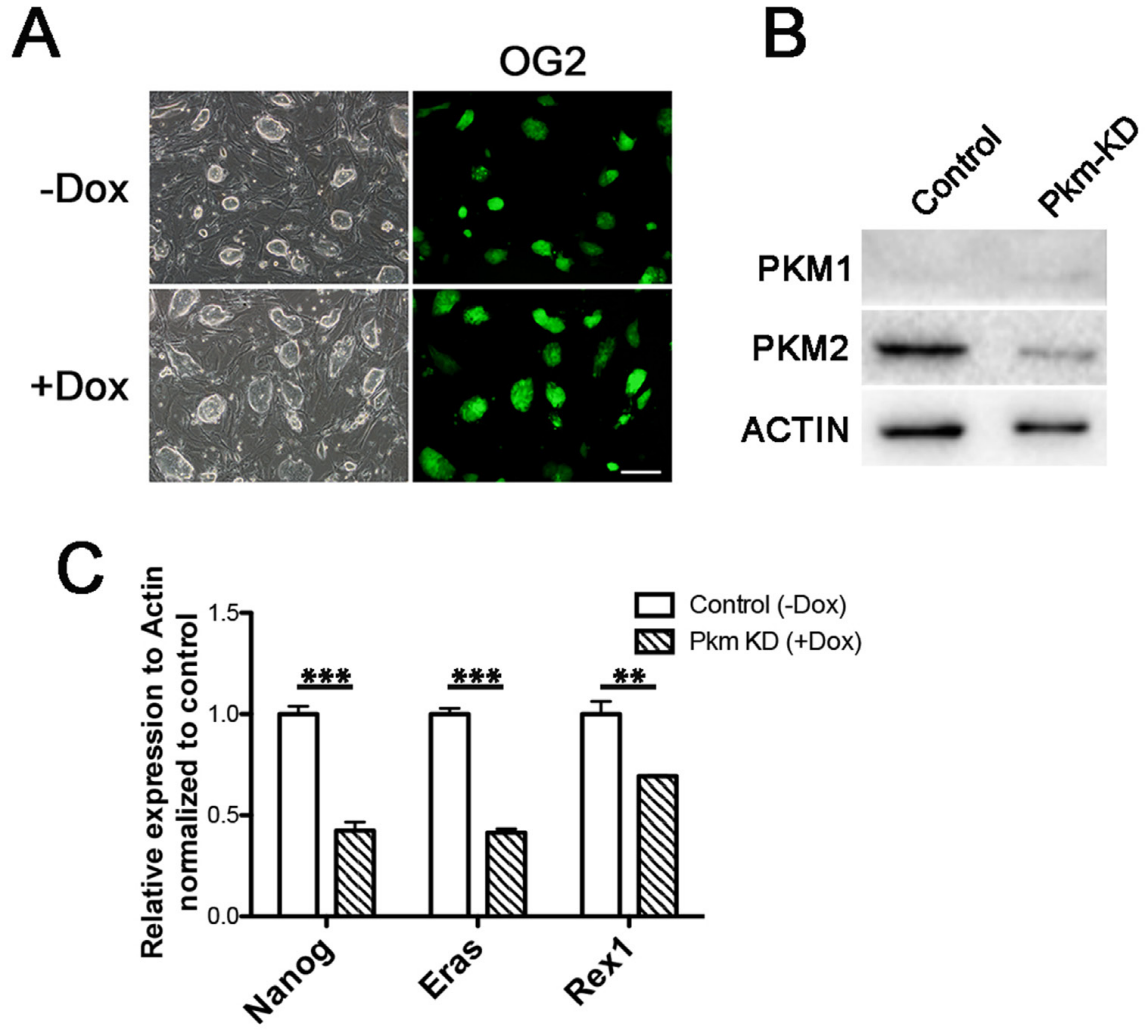
Knockdown of Pkm affected pluripotent genes **(A)** Morphology of the inducible Pkm knockdown ES cell lines without (upper) or with (lower) Dox addition. **(B)** Western blot showing the knockdown of Pkm1 and Pkm2 in established ES cell lines. **(C)** Real-time PCR examining the expression changes of Nanog, Eras and Rex1 under Pkm knockdown in ESCs. Scale bars represent 200 μm. Data represented as mean ± SD; n = 3.

Although Pkm2 could increase the expression of some pluripotent genes, the role of it in pluripotent cells seems not so crucial, as the availability of KO mice of Pkm2 has been reported previously [20]. We employed the rtTA-OG2 ESCs to establish the Pkm knockdown cell lines to elucidate the effect of Pkm defect in ESCs. Dox controlled inducible lentiviral overexpression vector carrying Cre recombinase was first introduced into the cells followed with the shRNA constructed in pSico. Under the Dox addition, expression of Cre could recombine out the EGFP sequence of the construct and then activate shRNA expression. We established stable cell lines with normal colony appearance and Oct4-EGFP positive signal (Figure 3A). The pluripotent genes we tested above also decreased under the shRNA mediated interference of Pkm in our established knockdown ESCs lines (Figure 3B and 3C). But the cell morphology and self-renewal didn't get obvious alteration (Figure 3A). Taken together with the result from overexpression study, Pkm1 and Pkm2 may interfere the pluripotent property of ESCs to some extent, but not crucial.

### Pkm2 was up-regulated and required in somatic cell reprograming

**Figure 4:**
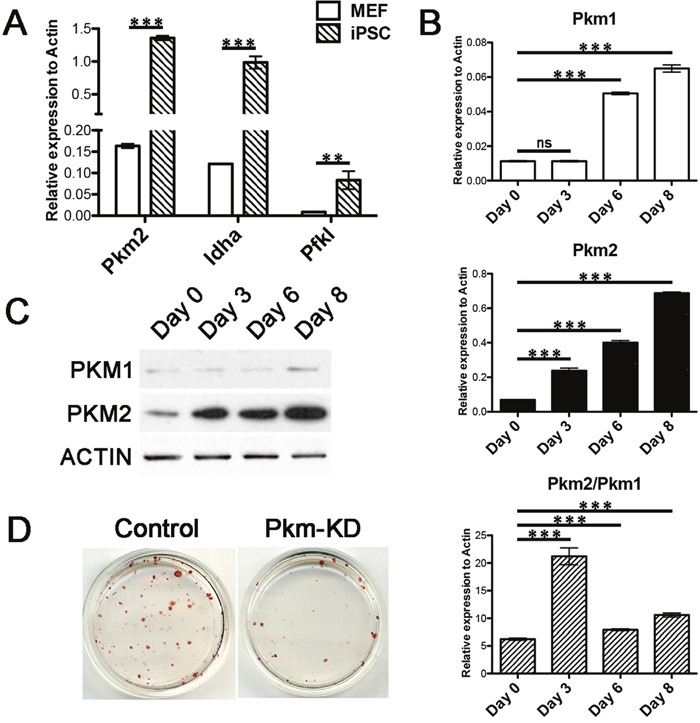
Pkm2 increased during reprogramming and was critical for iPS induction **(A)** Real-time PCR examining the expression of Pkm2, Idha and Pfkl in MEFs and ESCs. **(B)** Real-time PCR examining the expression of Pkm1, Pkm2 and their ratio at indicated time points during reprogramming. **(C)** Western blot showing the protein level of Pkm1 and Pkm2 at indicated time points during reprogramming. **(D)** Alkaline Phosphatase (AP) staining of iPSC colonies under Pkm knockdown. Data represented as mean ± SD; n = 3.

Based on the role of Pkm2 in pluripotency maintenance, we next aimed to investigate its function in pluripotency acquisition during somatic cell reprogramming. We employed the transgenic mice: Gt(ROSA)26Sor^tm1(rtTA^*^M2)Jae^ Col1a1^tm4(tetO-Pou5f1,-Sox2,-Klf4,-Myc)Jae^ (rtTA-OSKM) and Tg(Pou5f1-EGFP)2Mnn (OG2) to establish a secondary reprogramming system [21]. The somatic cells derived from the crossed offspring can be reprogrammed to iPSCs under the induced expression of genetically integrated Oct4, Sox2, Klf4 and c-Myc controlled by Dox. The success reprogramming can be detected by emergence of Oct4-EGFP positive iPSC colonies. As a rate-limiting enzyme in glycolysis, Pkm2 was found high expression in iPSCs compared with MEFs together with other important enzymes (Figure 4A). The RNA and protein level of Pkm1 and Pkm2 were increased during reprogramming, especially Pkm2, and the ratio of Pkm2 and Pkm1 was either getting higher (Figure 4B and 4C). It suggested an important role of Pkm in reprograming. Indeed, knocking down of Pkm by shRNA impaired the somatic reprograming greatly (Figure 4D).

### Overexpression of Pkm2 but not Pkm1 can facilitate somatic cell reprograming

**Figure 5:**
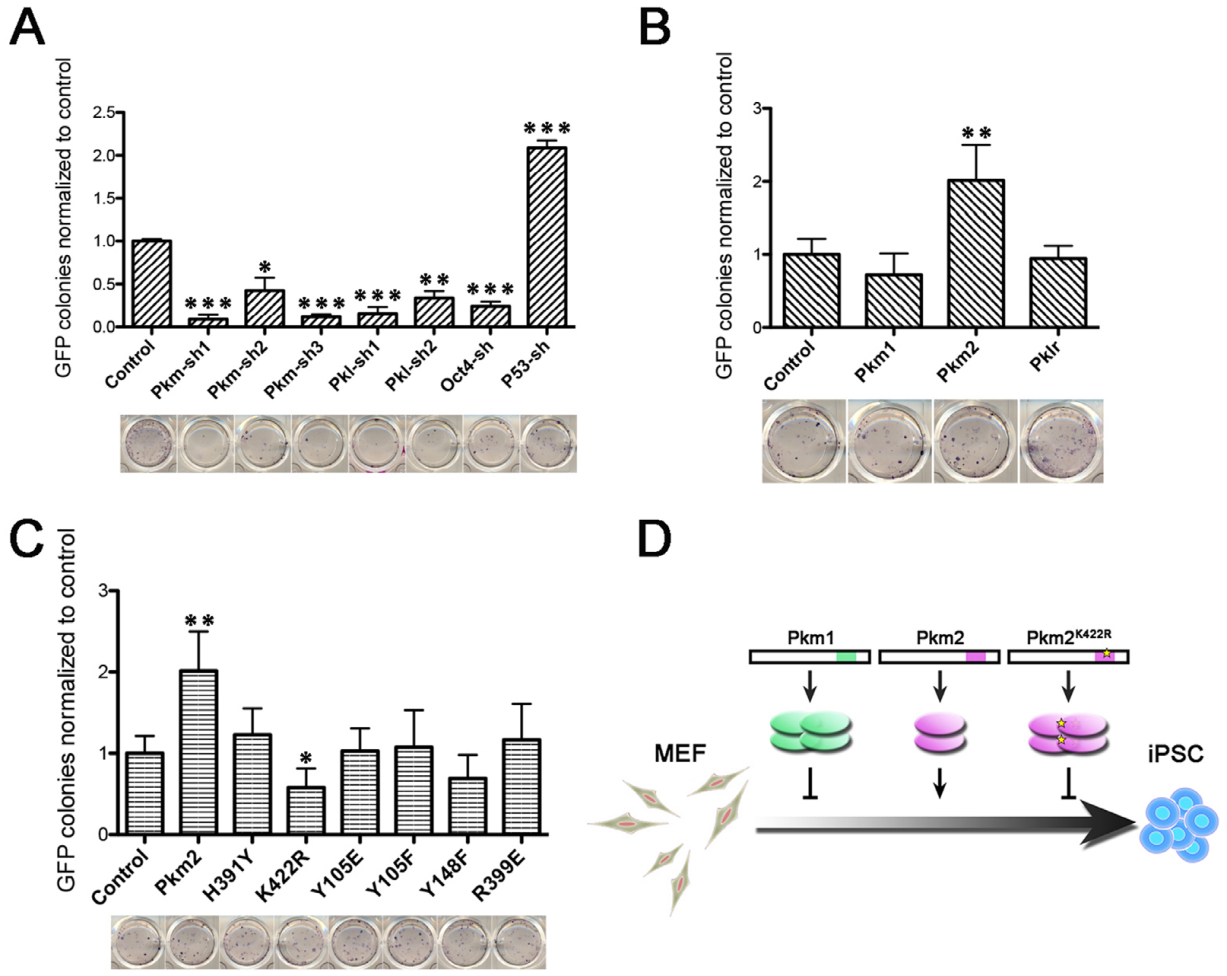
Pkm2 but not Pkm1 facilitated somatic reprogramming **(A)** Relative GFP^+^ colony number (upper) and AP staining (lower) of iPSC colonies that were formed under shRNA mediated knockdown of Pkm, Pkl, Oct4 or P53. shRNA of Oct4 and P53 served as negative and positive effector to reprogramming respectively. **(B)** Relative GFP^+^ colony number (upper) and AP staining (lower) of iPSC colonies that were formed under overexpression of Pkm1, Pkm2 or Pklr. **(C)** Relative GFP^+^ colony number (upper) and AP staining (lower) of iPSC colonies that were formed under overexpression of Pkm2 or indicated mutants. **(D)** Schematic diagram showing the conformation variance of Pkm1, Pkm2 and Pkm2^K422R^ and their different effects on reprogramming. GFP^+^ colony number of each sample was normalized to the control group. Data represented as mean ± SD; n = 3.

Different conformation, abundance and enzyme activity between Pkm1 and Pkm2 had been widely discussed in numerous tumor systems. Therefore, we next sought to explore the function of Pkm in somatic cell reprogramming. Using more shRNA sequences, we confirmed the negative effect on reprogramming of Pkm knocking down (Figure 5A). While the shRNA was unable to distinguish Pkm1 and Pkm2, we examined the overexpression effect of Pkm1, Pkm2 and Pklr on reprogramming. We found that Pkm2 which prior to exist as a dimer conformation with lower pyruvate kinase activity facilitated the iPSC induction while Pkm1 and Pklr didn't (Figure 5B). This suggested that the function of Pkm in reprogramming might correlated with its conformation and enzyme activity. So we employed the mutant forms of Pkm2 which had altered property [22, 23]. We found that the mutants retrieved the effect of Pkm2 on reprogramming, and the Pkm2^K422R^ even impaired iPSC induction efficiency (Figure 5C). It is understandable that Pkm2^K422R^ acts as a dominant tetramer conformation, which is not preferred in reprogramming. Thus, Pkm2 played positive role in somatic reprogramming which was correlated to its dimer conformation.

## DISCUSSION

It has been proposed that Pkm2 is playing important role in tumor progression, together with the switch from Pkm1 to Pkm2 being supposed to indicate the malignance in some tumors [11, 16]. So it turns important to elucidate its function in normal tissues especially in pluripotent stem cells (PSCs), which shares many properties with tumor cells. As we showed, overexpression or knockdown of Pkm2 influenced expression of some pluripotent genes coincident with previous finding that Pkm2 increased in the hypoxia culture of human ESCs and regulated Oct4 expression [24]. As to reprogramming, which is a process of transition from somatic cells to PSCs imitating tumor progression to some extent, the switch from Pkm1 to Pkm2 can hardly be proposed. Although the ratio of Pkm2 to Pkm1 raised after the reprogramming, it seemed mainly due to the increase of Pkm2 while Pkm1 barely expressed in the whole process.

As the predominant pyruvate kinase in ESCs, Pkm2 not only increased along with reprogramming, but also facilitate this process. Besides the tetrameric quaternary structure like Pkm1, which has high affinity to PEP, Pkm2 also has dimeric form with low affinity to PEP. In tumor cells, Pkm2 was found mainly in dimeric form, leading to the accumulation of glycolytic intermediates, and thus providing amount of building blocks for synthetic processes, which are badly needed by highly proliferating cells. It has also been shown that the dimeric Pkm2 not only serves as pyruvate kinase participates in metabolism but also translocates into nucleus and cooperates with other proteins [25, 26]. Supporting these reports, Pkm2 rather than Pkm1 could advance the iPSCs induction, which is coincident with the effect of Hif1-α on reprogramming [27]. Further more, this effect could be reverted by site mutation K422R, which converts the conformation of Pkm2 to tetrameric structure (Figure 5D). So, it is the specific property of dimeric form facilitates reprogramming just like in cancer progression.

It was interesting to find that Pkm2 deletion accelerated tumor formation rather than repress it [20, 28]. Proliferating and nonproliferating tumor cells exhibited different requirement for Pkm1 and Pkm2 [20], which might offer a good explanation for the controversy in cancer research mentioned above. The function and regulation of Pkm2 might vary depending on different cell types and processes. Ptb and Hnrnp, which have been shown to increase the Pkm2/Pkm1 ratio in tumor cells [16], did not have obvious effect on increasing Pkm2 level nor iPSC colony number in reprogramming (data not shown). Thus, further exploration of the genes affecting Pkm2's expression in reprogramming might offer new aspects for its regulation mechanism and in turn help to understand its role in tumor.

## MATERIALS AND METHODS

### Animals

Gt(ROSA)26Sor^tm1(rtTA^*^M2)Jae^ Col1a1^tm4(tetO-Pou5f1,-Sox2,-Klf4,-Myc)Jae^ and Tg(Pou5f1-EGFP)2Mnn transgenic mice were employed in this study.

Specific pathogen-free mice were housed in the animal facility of Dalian Medical University. All studies adhered to procedures that were consistent with the Dalian Medical University Guide for the care and use of laboratory animals.

### ESCs maintenance and iPSCs generation

ESCs and iPSCs were cultured on mitomycin C treated MEFs in ESC culture medium composed with DMEM (Merk Millipore) supplemented with 15% (v/v) fetal bovine serum (Hyclone), 1 mM L-glutamine (Merk Millipore), 0.1 mM mercaptoethanol (Invitrogen), 1% nonessential amino acid stock (Merk Millipore), nucleosides (100×, Merk Millipore) and 1000 U/ml LIF (Merk Millipore).

For iPSCs induction, MEFs were derived from Gt(ROSA)26Sor^tm1(rtTA^*^M2)Jae^ Col1a1^tm4(tetO-Pou5f1,-Sox2,-Klf4,-Myc)Jae^ crossed with Tg(Pou5f1-EGFP)2Mnn transgenic mice. After culturing in ESC culture medium containing Dox for 12 days, ESC-like colonies appeared, and then the Dox was removed from the culture medium. ESC-like colonies were individually digested and replated. After propagation, we selected iPS cell lines that exhibited typical ES cell morphology for long-term culture.

### Embryoid body (EB) formation

ESCs were seeded in the culture plate with Ultra-low attachment surface (Corning) in differentiation medium composed with DMEM (Gibco) supplemented with 10% (v/v) fetal bovine serum (Hyclone), 1 mM L-glutamine (Merk Millipore), 0.1 mM mercaptoethanol (Invitrogen), 1% nonessential amino acid stock (Merk Millipore), nucleosides (100×, Merk Millipore). After 5 days, the EBs were transferred to the gelatin coated cell culture dish for further differentiation.

### Quantitative detection for indicated genes

Total RNA was extracted using TRIzol reagent (Invitrogen) and reverse transcribed by 5×All-In-One RT MasterMix (Abm). Real-time PCR was performed with KAPA SYBR fast qPCR kit on the Real-time PCR System (Applied Biosystems). β-Actin served as internal control.

Pkm1-F: GCCTCCAGTCACTCCACAGA, Pkm1-R: CAGCACGGCATCCTTACACA; Pkm2-F: CAGCACCT GATTGCCCGAGA, Pkm2-R: CCAGACTTGGTGAGCAC GATA; Pklr-F: CTATGGCGGACACCTTCC, Pklr-R: TGT TCATCCCTGCCTTGAT; Nanog-F: CACCCACCCATG CTAGTCTT, Nanog-R: ACCCTCAAACTCCTGGTCCT; Eras-F: ACTGCCCCTCATCAGACTGCTACT, Eras-R: CA CTGCCTTGTACTCGGGTAGCTG; Rex1-F: ACGAGTGG CAGTTTCTTCTTGGGA, Rex1-R: TATGACTCACTTCC AGGGGGCACT; Oct4-F: CTGAGGGCCAGGCAGGA GCACGAG, Oct4-R: CTGTAGGGAGGGCTTCGGG CACTT; Fgf5-F: AACTCCATGCAAGTGCCAAAT, Fgf5 -R: CGGACGCATAGGTATTATAGCTG; Afp-F: CTTC CCTCATCCTCCTGCTAC, Afp-R: ACAAACTGGGTAAA GGTGATGG; Gata6-F: TTGCTCCGGTAACAGCAGTG, Gata6-R: GTGGTCGCTTGTGTAGAAGGA; Actin-F: AGAGGGAAATCGTGCGTGAC, Actin-R: CAATAG TGATGACCTGGCCGT.

### Western blot and antibodies

The cells were harvested, washed with cold PBS, and lysed with RIPA buffer. The lysates were centrifuged at 16,000 g for 10 minutes at 4°C to remove cell debris. Proteins was analyzed under denaturing conditions in 10% SDS-polyacrylamide gel electrophoresis (SDS-PAGE), and transferred onto polyvinylidene difluoride (PVDF) membranes. Blots were blocked in TBST buffer supplemented with 5% skim milk for 1 hour, followed by probing with primary antibodies 4°C overnight. After three washes with TBST buffer, the blots were incubated with secondary antibodies conjugated to HRP in TBST buffer for 1 hour, followed by three washes with TBST buffer. ECL Prime Western Blotting detection reagent (GE Healthcare) was used to generate chemiluminescence signals which were detected by Chemi Doc Touch imaging system (Bio-Rad). ß-ACTIN was used as a loading control.

The antibody against PKM2 was from Cell Signaling Technology. PKM1 antibody was from Proteintech. ß-ACTIN anbibody was from Transgen Biotech.

### Alkaline phosphatase (AP)

Alkaline Phosphatase staining was performed according to the manufacturer's recommendation using the Alkaline Phosphatase Detection Kit (Millipore).

### Statistical analysis

Each experiment was performed with a minimal of three biological replicates and the replicate number is given in the figure legends. Mean and standard deviation were calculated as indicated. Statistical analyses used unpaired, Student's t tests to test significance. Where indicated, ns = not significant, ^*^ p ≤ 0.05, ^**^ p ≤ 0.01, ^***^ p ≤ 0.001.
